# A second generation genetic linkage map of Japanese flounder (*Paralichthys olivaceus*)

**DOI:** 10.1186/1471-2164-11-554

**Published:** 2010-10-11

**Authors:** Cecilia Castaño-Sánchez, Kanako Fuji, Akiyuki Ozaki, Osamu Hasegawa, Takashi Sakamoto, Kagayaki Morishima, Ichiro Nakayama, Atsushi Fujiwara, Tetsuji Masaoka, Hiroyuki Okamoto, Kengo Hayashida, Michihira Tagami, Jun Kawai, Yoshihide Hayashizaki, Nobuaki Okamoto

**Affiliations:** 1 Faculty of Marine Science, Tokyo University of Marine Science and Technology, Minato, Tokyo, Japan; 2 National Research Institute of Aquaculture, Fisheries Research Agency, Watarai, Mie, Japan; 3 Kanagawa Prefectural Fisheries Technology Center, Miura, Kanagawa, Japan; 4 National Research Institute of Fisheries Science, Fisheries Research Agency, Yokohama, Kanagawa, Japan; 5 RIKEN Omics Science Center, RIKEN Yokohama Institute, Yokohama, Kanagawa, Japan

## Abstract

**Background:**

Japanese flounder (*Paralichthys olivaceus*) is one of the most economically important marine species in Northeast Asia. Information on genetic markers associated with quantitative trait loci (QTL) can be used in breeding programs to identify and select individuals carrying desired traits. Commercial production of Japanese flounder could be increased by developing disease-resistant fish and improving commercially important traits. Previous maps have been constructed with AFLP markers and a limited number of microsatellite markers. In this study, improved genetic linkage maps are presented. In contrast with previous studies, these maps were built mainly with a large number of codominant markers so they can potentially be used to analyze different families and populations.

**Results:**

Sex-specific genetic linkage maps were constructed for the Japanese flounder including a total of 1,375 markers [1,268 microsatellites, 105 single nucleotide polymorphisms (SNPs) and two genes]; 1,167 markers are linked to the male map and 1,067 markers are linked to the female map. The lengths of the male and female maps are 1,147.7 cM and 833.8 cM, respectively. Based on estimations of map lengths, the female and male maps covered 79 and 82% of the genome, respectively. Recombination ratio in the new maps revealed F:M of 1:0.7. All linkage groups in the maps presented large differences in the location of sex-specific recombination hot-spots.

**Conclusions:**

The improved genetic linkage maps are very useful for QTL analyses and marker-assisted selection (MAS) breeding programs for economically important traits in Japanese flounder. In addition, SNP flanking sequences were blasted against *Tetraodon nigroviridis *(puffer fish) and *Danio rerio *(zebrafish), and synteny analysis has been carried out. The ability to detect synteny among species or genera based on homology analysis of SNP flanking sequences may provide opportunities to complement initial QTL experiments with candidate gene approaches from homologous chromosomal locations identified in related model organisms.

## Background

Genetic linkage maps have been developed for several species. Microsatellite markers have been commonly chosen in linkage maps because they exhibit codominant inheritance, have high degrees of heterozygosity, are widely distributed throughout the genomes, and provide comparative information between closely related species [1]. In aquaculture species, first generation, low resolution genetic linkage maps have been developed for many species, including tilapia (*Oreochromis niloticus*) [2,3], channel catfish (*Ictalurus punctatuts*) [4], rainbow trout (*Oncorhynchus mykiss*) [1,5], Atlantic salmon (*Salmo salar*) [6], brown trout (*Salmo trutta*) [7], European sea bass (*Dicentrarchus labrax *L.) [8], gilthead sea bream (*Sparus aurata *L.) [9], turbot (*Scophthalmus maximus*) [10], Atlantic halibut (*Hippoglossus hippoglossus *L.) [11], half-smooth tongue sole (*Cynoglossus semilaevis*) [12], pacific abalone (*Haliotis discus hannai*) [13,14] and oyster (*Crassostrea gigas*) [15,16]. Recently, second generation maps that span the genomes at higher resolution have been constructed. These maps contain several hundred of markers with microsatellites and single nucleotide polymorphisms (SNPs) associated with candidate genes. They have been constructed for tilapia [17], rainbow trout [18,19], Atlantic salmon [20]and channel catfish [21].

Japanese flounder, *Paralichthys olivaceus*, is widely distributed along the coast of Northeast Asia and is one of the most economically important marine species in the region. *P. olivaceus *is successfully cultured in Japan, China and Korea. Like other aquaculture species, Japanese flounder is susceptible to several viruses, bacteria and protozoan pathogens, and may also show pigmentation abnormalities, which decrease its market price [22]. Some economically important traits like disease resistance and growth are quantitative phenotypes and their genetic basis relies on the combined effects of quantitative trait loci (QTL) [23]. Information on genetic markers associated with QTL can be used in marker-assisted selection (MAS) breeding programs to identify and select individuals carrying desired traits. Commercial production of Japanese flounder could be increased by developing disease-resistant fish and improving commercially important traits. Fuji *et al. *[24,25] found a single major genetic locus associated with lymphocystis disease resistance in Japanese flounder and succeeded in commercially producing a lymphocystis disease-resistant strain by marker-assisted selection (MAS).

Linkage maps are essential tools to study QTL, therefore, sex-specific genetic linkage maps were first constructed for Japanese flounder by Coimbra *et al. *[26] included a total of 111 di-nucleotide microsatellite markers and 352 AFLP fragments and contained thirty linkage groups. That map was arbitrary named BA map, since it was based on a family bred out of strains "KP-A" and "KP-B" from Kanagawa prefecture fisheries technology center. Linkage maps need to be built mainly with codominant markers, which are representative of the same loci across studies. Maps built with codominant markers can be used in different families and populations. Therefore, new sex-specific maps (named A2 maps) were constructed using 230 di-nucleotide microsatellite [27]. Markers in those maps were distributed in 24 linkage groups; the number of linkage groups was in accordance with the haploid chromosome number of the Japanese flounder [28]. Moreover, gynogenetic diploids derived from the dam of the A2 map were used to estimate the centromeric regions in the map. Additionally, a sex-averaged map was constructed based on 180 microsatellites from BA maps as well as other previously isolated markers and 31 newly developed EST-derived microsatellites [29].

Haldane [30] established that when meiotic recombination rates vary between sexes it is usually the heterogametic sex that has lower recombination. The sex with lower recombination rates is expected to transmit marker-QTL associations in tighter linkage. Thus, the study of differences in recombination rates between sexes could be of great importance in MAS programs [26]. Though the sex determination systems of fish are variable, most fish species for which linkage maps have been developed exhibit male heterogamety. In these species, linkage mapping indicates that recombination rates in males are greatly reduced compared to females [10,11]. However, in a preliminary study of Japanese flounder, males appeared to have higher recombination rates than females [26]. In this study, further analyses were performed using a larger number of markers. These analyses were performed with segregating data of the A2 family used for a construction of the A2 map, as well as the BACE family of the improved genetic linkage map. This family was a hybrid from four strains from the Kanagawa Prefecture Fisheries Center, strains "KP-A", "KP-B", "KP-C" and "KP-E" [24] and has been named BACE.

In this report, we present significantly improved female and male maps (BACE map), which contain 1,375 markers, including microsatellites, SNPs (Single Nucleotide Polymorphisms) and two genes. These maps will facilitate the genome mapping efforts in Japanese flounder and other related species. The mapping data could be compared to reference species and utilized for QTL analyses and further MAS.

## Results

### Microsatellite markers

Hybridization and sequencing results detected 5,930 positive clones, containing 7,791 microsatellite regions. A total of 1,808 primer pairs were designed, and polymorphism was checked; 34% of the clones were polymorphic in both parents of the BACE family and 21% of the clones were polymorphic in either the male or female of the family. Among those markers, 746 microsatellites were selected and genotyped in the BACE family. Sequence data of the newly developed microsatellites have been deposited with the GenBank Data Library under the accession nos: EF112585-EF113072 and AB458899-AB459282. A list of the 1,268 microsatellite markers included in the map is presented in additional file 1.

### SNP markers

Seventeen new "AB" SNP marker, derived from new EST sequences of Japanese flounder were polymorphic in either the male or female of the BACE family and were included in the map (Additional file 2). They are represented in the BACE map with their respective GenBank accession numbers; AB275460, AB275461, AB275463-AB275466 and AB275468-AB275478. The analysis of the EST sequences obtained from the NCBI database resulted in 88 polymorphic SNP markers (Additional file 2).

### Markers in the BACE map

**Figure 1:**
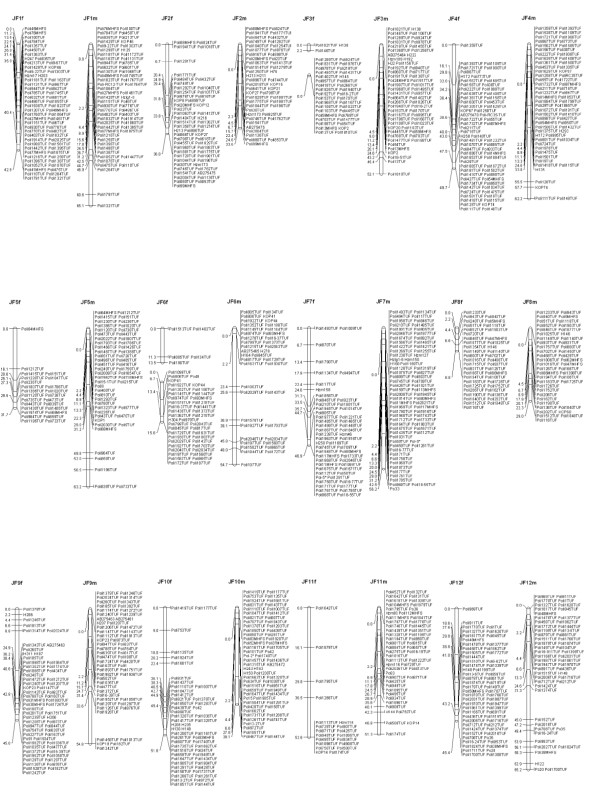
**Japanese flounder male (left) and female (right) maps, Linkage groups JF1 - JF12**. Total lengths of linkage groups are expressed in Kosambi cM. Assigned names of loci and linkage groups are consistent with the map published by Coimbra *et al. *2003. Microsatellites are coded "*Poli" *followed by a number and the laboratory designation (TUF, MHFS). SNP markers are labeled either with their respective GenBank accession numbers or with the letters "H" and "Hzm" followed by their corresponding number.

**Figure 2:**
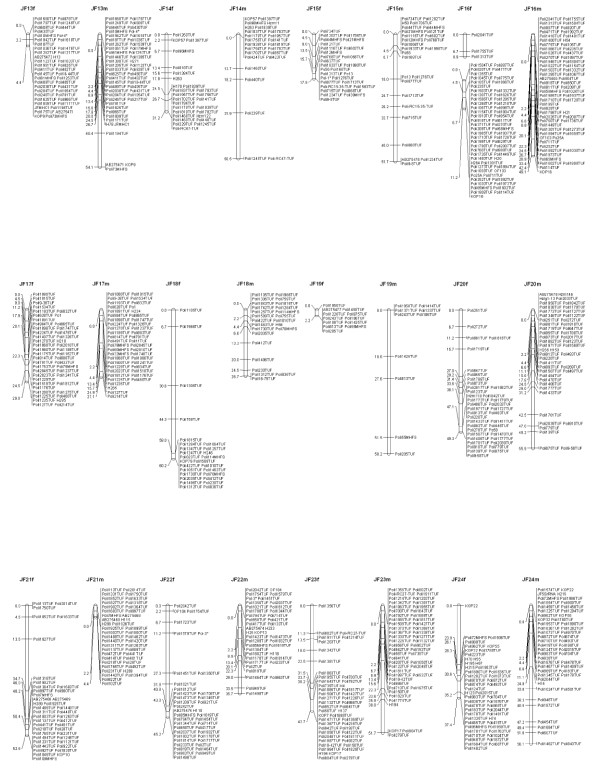
**Japanese flounder male (left) and female (right) maps, Linkage groups JF13 - JF24**. Total lengths of linkage groups are expressed in Kosambi cM. Assigned names of loci and linkage groups are consistent with the map published by Coimbra *et al. *2003. Microsatellites are coded "*Poli" *followed by a number and the laboratory designation (TUF, MHFS). SNP markers are labeled either with their respective GenBank accession numbers or with the letters "H" and "Hzm" followed by their corresponding number. The Poli9-8TUF in JF15f is associated with lymphocystis disease resistance of Japanese flounder, reported by Fuji *et al. *(2007).

A total of 1,375 markers including 1,268 microsatellites, 105 SNPs and 2 genes were mapped in sex-specific linkage groups. The male map contains 1,167 markers and the female map contains 1,067 markers (Figures 1 and 2; additional files 3, 4 and 5). In accordance with the 24 haploid chromosome number of the families confirmed by a karyotype study [28], markers were distributed along 24 linkage groups. The chromosomes of *P. olivaceus *are all acrocentric [28] and centromeric regions have been estimated by centromere-mapping using gynogenetic diploids derived from a dam [27]. The linkage groups in Figures 1 and 2 are represented with their predicted centromeric regions from the top of the group.

Previous versions of the BA and A2 maps were built with only di-nucleotide microsatellites. The BACE map includes di-, tri-, and tetra-nucleotide microsatellite markers as well as SNP markers. As a general rule, the different kinds of markers appeared to be equally distributed along the linage groups. The microsatellite locus derived from the sequence of the MHC class Ia genes in *P. olivaceus *was linked to JF13. By means of fluorescence *in situ *hybridization (FISH), the locus in JF13 was identified in chromosome 17 among Japanese flounder karyotypes characterized by C-band staining (data not shown). MHC genes play an important role in the immune system. The genetic information developed in this work, together with the information of its location on chromosome 17, could be of importance in the study of disease resistance. Furthermore, other immune-related genes that have already been isolated for this species, like the immunoglobulin genes [31] could be included in the map. In addition, the 5 S rRNA gene was only polymorphic for the sire in the BACE family and was placed in the linkage group JF24 m. By *in situ *hybridization, this gene was reported to be physically located on chromosome 1 [28].

### Map length and coverage

The total length of the male map is 1,147.7 cM. Linkage group sizes ranged from 4.4 cM (JF21m) to 65.2 cM (JF12m). The female map spanned 833.8 cM. JF19f is unexpectedly short (2.2 cM) and the longest linkage group (JF18f) extends to 60.2 cM. The maps contained several co-segregating loci. Thus, the average resolution of the maps was estimated by collapsing those loci into "bins" and calculating the average inter-marker distances for all adjacent "bins" and single markers (framework markers). The male and female maps had 235 and 184 unique positions, respectively, with average intervals of 5.0 cM. and 4.4 cM, respectively. The average estimated genome size of the male map (1,394.2 cM) is longer than the speculated 1,155 cM length found by Coimbra *et al. *[26]. On the other hand, the female map (1,055.7 cM) is in closer agreement with the length found by Coimbra *et al. *[26] (1,176 cM). Based on recent estimations of map lengths, the genome coverage of the male and female maps were 82% and 79%, respectively. The male map is 1.3 times longer than the female map. However, this difference is not consistent along all linkage groups. Some groups (JF10; JF18; JF21; JF22) are longer in the female map (Figures 1 and 2). A similar pattern was observed in turbot [10] and Atlantic halibut [11].

**Figure 3:**
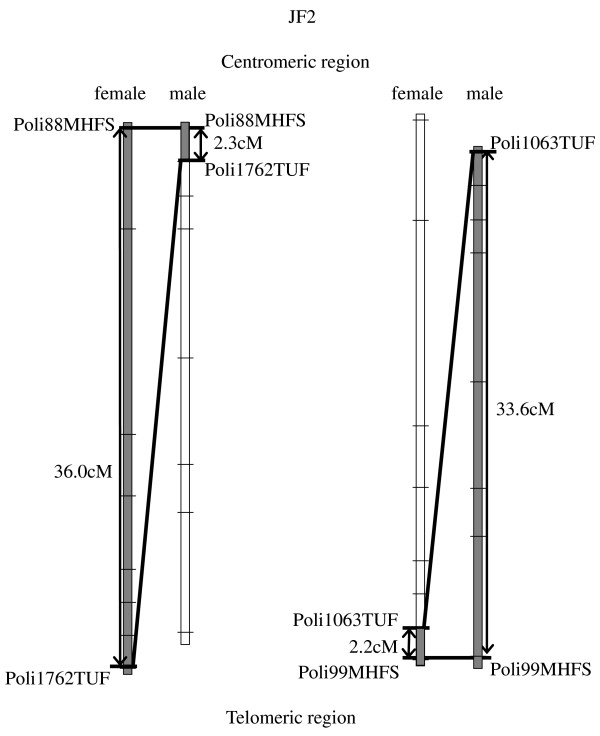
**Evidences of differences in recombination regions in male and female maps, Linkage group 2 as an example**. The distance from marker "Poli88MHFS " placed in the bin closer to the estimated centromeric region in the male map, to marker "Poli1762TUF", in the second bin, is 2.3 cM. However, in the female map, marker "Poli1762TUF" locates at 36.0 cM from the first bin from the centromeric region. Conversely, .marker "Poli1413TUF" is located at 2.2 cM from the closest bin ("Poli99MHFS") from the telomeric region, but it is 33.6 cM apart in the male map.

Over the entire length of the linkage groups, the distribution of the markers is not uniform. All linkage groups tend to be compressed in the estimated centromeric region in the male map and in the telomeric region in the female map (Figure 3). In females, a higher rate of recombination occurred in the centromeric regions across linkage groups. On the other hand, in males, a higher rate of recombination was observed in the telomeric regions. Accordingly, a higher clustering of markers is observed in the telomeric regions of female maps and near the centromeric regions of male-specific linkage groups. Despite the extremely large differences in the chromosomal localization of the sex-specific recombination spots in Japanese flounder, when averaged over all markers in the genetic map, only small difference in female:male recombination ratios was observed (i.e., F:M ratio 1:0.7).

### Annotation and synteny

A hundred and five ESTs showed high similarity to known protein (*E*-values < 1 × 10^4^). Thirty-two of these ESTs significantly matched (*E*-values < 1 × 10^-4^) sequences against *T. nigroviridis*, and 35 also matched *D. rerio *sequences (Additional file 6). Sixteen linkage groups (JF LGs: Japanese flounder Linkage Groups) in the BACE map could be associated with *T. nigroviridis *chromosomes, and nineteen JF LGs with *D. rerio *chromosomes. Among JF LGs with at least two ESTs, seven JF LGs (JF1, JF7, JF10, JF11, JF14, JF21, JF24) were syntenic with individual *T. nigroviridis*n chromosomes, five of them (JF1, JF10, JF20, JF21, JF24) were also syntenic with individual *D. rerio *chromosomes and JF1, JF10 and JF24 were associated to a single chromosome of both *T. nigroviridis *(Chromosomes 9, 8 and 3, respectively) and *D. rerio *(Chromosomes 22, 16 and 3, respectively). ESTs in JF3, JF9, JF11 and JF16 were associated with different chromosomes of *D. rerio *(JF3: Chromosomes 9 and 23, JF9: Chromosomes 22, 6 and 2, JF11: Chromosomes 25, 18 and 19, JF16: Chromosomes 6 and 11). No ESTs in a single JF LG were associated with more than one chromosome of *T. nigroviridis*. Two *T. nigroviridis *chromosomes (Nos. 2 and 3) and 6 chromosomes (Nos. 2, 6, 9, 16, 22 and 23) of *D. rerio *were associated with ESTs from different linkage groups of the JF map (Additional file 7).

## Discussion

Recombination events in fish species usually occur once per chromosome arm, indicating the existence of interference after the formation of a single chiasma [32]. Japanese flounder is a male-determined gonochoristic species [33] and presents the same pattern of recombination as other fish species, in which the male presents a higher recombination rate closer to the putative telomere, and the female presents a higher recombination rate closer to the centromere. Evidence of this phenomenon was reported in rainbow trout [1]; brown trout [7], zebrafish [34] and Atlantic halibut [11]. The A2 map [27] and BACE map of Japanese flounder present the same pattern of recombination as other fish species. Figure 3 is a schematic representation of linkage group 2, and shows a clear example of this phenomenon, where a set of markers (bin) is separated by 2.3 cM from the centromeric region in the male map and 36.0 cM in the male. In contrast, the first recombination point in the female map is located at 2.2 cM from telomeric regions and 33.6 cM in the male map. Even though the lengths of the female and male maps were similar, Coimbra *et al. *[26] found an unexpected F:M 1:7.4. In that study, because the male was gynogenetically produced, it is unclear whether the ratio was influenced by the genetic origin of the male. In addition, only a few markers from 16 linkage groups were used to perform the analyses. The actual positions of those markers in the chromosomes were determined in the marker-centromere map built by Castaño-Sánchez *et al. *[27]. All marker pairs analyzed by Coimbra *et al. *[26]were placed far from the estimated centromeric regions, which are characterized by less recombination activity in the Japanese flounder male map and higher recombination rates in the female map. Reid *et al. *[11] reported the recombination rate in female Atlantic halibut to be twice that of the male, and observed a significant difference of F:M 1.6:1. Analysis of overall recombination rates between males and females in the BACE map confirmed a F:M ratio of 1:0.7. The improved male map is 1.4 times longer than the female map. Conversely, the male sheep map *(Ovis aries) *is 1.2 times longer than the female map and cattle *(Bos taurus) *maps present a very similar rate between sexes [35,36]. While all chromosomes in Japanese flounder are acrocentric, the cattle karyotype contains 29 acrocentric autosomes and the sheep has 23 acrocentric and 3 metacentric autosomes [36]. In humans, there is evidence that recombination along the chromosomes depends on the chromosome structure [37]. The presence of acrocentric karyotypes in sheep and Japanese flounder could explain the fact that the male map is slightly longer than the female map in those species, and accordingly, similar in length in the cattle map. These findings, together with the reported existence of gaps longer than 20 cM between adjacent markers in some linkage groups, might indicate poor coverage in certain regions of the female map. Incomplete female maps might reflect that a higher proportion of crossovers in female generated maps will be missed, causing an underestimation of recombination rates in females relative to males, and therefore artificially decreasing F:M recombination ratios.

Synteny among species or genera may provide opportunities to complement initial QTL experiments with candidate gene approaches from homologous chromosomal locations identified in related model organisms [38]. Based on the sequence homology analysis, more of Japanese flounder chromosomes were associated with *T. nigroviridis *chromosomes than *D. rerio *chromosomes; accordingly, Japanese flounder is phylogenetically more closely related to *T. nigroviridis *than to *D. rerio *[39]. In addition, analysis results suggest that, during evolution, some chromosomes and regions have remained intact and others have been broken up. Ancient Actinopterygii (ray-finned fish) were postulated to have a 13 chromosome karyotype, composed of 52 A'-J' segments. Those blocks were mosaically arrayed within the proto-Actinopterygian karyotype and subsequently designated A-M (reviewed by [20]). Based on Danzmann *et al. *[20], the association of JF9 to *D. rerio *chromosomes Dr2, 6 and 22 and JF16 with Dr6 and 11, might indicate a relation of those linkage groups to "M" ancestral grouping of Actinopterygians. Moreover, the association of JF11 to Dr18, 19 and 25 might suggests its relation to the "J" ancestral linkage groups, while JF3 is associated with Dr9 and 23 and could be related to either ancestral "C" or "L" lineages, being "L" more likely. This data indicates that those linkage groups are likely remnants of regions that share a high degree of 3R duplicated segments.

Low-density genetic linkage maps have been published for *P. olivaceus *[26,27,29]. The map developed in the present study was built with 1,375 markers including 1.268 microsatellites, 105 SNPs and two genes, which makes it more portable to other strains and families. This facilitates its application to QTL analyses as well as comparative mapping to reference animals. The average inter-marker distances (5.0 cM and 4.4 cM in the male and female maps, respectively) offer sufficient marker density for QTL studies [23].

The improved maps, in addition to being useful for improving aquaculture strains, could be of assistance in the study of wild stocks in Japan, where cultured *P. olivaceus *are being released into the wild. Maintaining genetic variability is essential for the conservation of the species, not only to prevent inbreeding and bottleneck effects, but also to protect the genetic structure of natural stocks. Several microsatellite markers included in the improved maps have been previously used in population studies, genetic tagging, parentage determination and genetic diversity [40-44].

With 1,375 markers, the new map is presently the densest flatfish linkage map. The number of genetic markers available for other flatfish species is relatively limited. In this report, we describe the production of a large number of polymorphic microsatellite markers for *P. olivaceus *which could be amplified in other closely related species. Japanese flounder markers have already been used in the construction of the Atlantic halibut linkage map [11]. Despite the limited number of comparison points between Japanese flounder and Atlantic halibut, Reid *et al. *[11] found evidences of conserved syntenic regions as well as regions of chromosome rearrangements. The markers mapped in this study, could be an important tool for future comparative map studies and to establish the correspondence between linkage groups of different flatfish species.

The microsatellite markers included in previous versions of the map [26,27,29] were consistently assigned to the same linkage groups in the newly developed maps. The order of those markers was conserved in most of the linkage groups. However, several markers co-segregate in clusters, preventing the determination of their precise order. Several regions in the maps remain poorly covered. JF19 in both female and male maps is short and has only a small number of markers. The A2 map presented several gaps, which tended to occur towards the putative telomere in the male map and centromere in the female [27]. By adding more markers, several gaps were filled, but there is still the need to improve the centromeric regions of some linkage groups in the female map (Figure 1: JF3f, JF6f, JF16f). Further studies with segregating data from different families and larger number of progeny will be necessary to enhance the distribution of the markers in the linkage maps. Physical maps could be constructed based on an existing BAC (Bacterial Artificial Chromosome) library [45] and they could be useful to determine the precise distribution and order of the markers in the genome.

## Conclusion

The new high density genetic linkage map of Japanese flounder indicated large differences in the location of sex-specific recombination hot-spots and produced comparative results against *T. nigroviridis *and *D. rerio *in synteny. This map could be of extreme importance for QTL analysis and MAS breeding programs for economically important traits in Japanese flounder as well as for comparative studies for related species and/or model fishes. The linkage maps have already been used in a MAS breeding project to increase lymphocystis disease resistance [24]. The map is currently being used in QTL studies of Edwardsiellosis, an infectious disease caused by *Edwardsiella tarda*. QTL data will be eventually used to develop a new strain of Japanese flounder with complex disease resistant traits.

## Methods

### Reference family

The improved maps were created using a hybrid population (BACE family) generated by a cross between four strains of *P. olivaceus *that had been bred for several generations at Kanagawa Prefectural Fisheries Technology Center. The dam (KP-BA) is a hybrid between strains KP-B and KP-A used in the linkage mapping and disease QTL studies of [26] and Fuji *et al. *[24], and the sire (KP-CE) derives from the strains KP-C (used for pseudo albinism studies, unpublished) and KP-E (more recently domesticated). The map was constructed by genotyping the parents and 45 of their F_1 _offspring.

### Microsatellite markers

To develop resources for linkage mapping, two small insert DNA libraries had been previously constructed by digesting genomic DNA with either *Tsp*509I or *Sau*3AI and approximately 200,000 recombinant clones were placed in high density filter membranes [46]. The filters were screened for microsatellites with a radiolabeled (CA)_10 _probe, following the procedures previously described. Hybridization and washing were performed at 50°C. Clones putatively containing microsatellites were sequenced and those containing perfect, imperfect or compound repeats with at least 10 repeats in length were chosen for primer design. When more than one microsatellite region was found in the same clone, only one region was used; redundant sequences were discarded. Primer (20-27mer) pairs flanking the microsatellite regions were designed using Primer3 [47]. All primers were designed for a 62°C annealing temperature, a total amplicon size of 100-150 bp and 45-60% GC content. PCR reactions (total volume of 12 μl) contained 50 ng of genomic DNA; 0.7 pmol of forward primer; 0.3 pmol of reverse primer end labeled with γ-^33^P [ATP]; 0.5 U of *Taq *polymerase; 1× PCR buffer; 100 μM of each dNTP and 1% BSA. Thermal cycles were carried out as follows: initial denaturing step (2 min at 95°C); 35 cycles (95°C for 30 s, 1 min at 62°C, 72°C for 1 min) and a final extension step (3 min at 72°C). PCR products were electrophoresed on 6% acrylamide gels and were identified with a Bio-Image Analyzer (Fujifilm Co.). Marker polymorphisms were checked using DNA of eleven individuals of five different families. Seven hundred forty-six microsatellites that proved to be polymorphic for the map family were genotyped. Mapping data was obtained by visual scoring of autoradiograms.

In addition, 522 microsatellites that were previously published or registered were used for a high density map (BACE map). A set of 164 tri- and tetra-nucleotide microsatellites isolated by Castaño-Sánchez *et al. *[46] were included in the map (DQ888908-889074), as well as other loci previously cloned in this laboratory [48]. The map also included 184 markers from the previous maps (BA and A2), 124 new markers that had not been mapped in the previous maps (GenBank accession no; DQ865460-865479, DQ868392, EF112607-112700, AB459284-459473) and 50 markers that were developed and genotyped by other authors [29,40-42,44,49].

### Genes

Major histocompatibility complex (MHC) class Ia, which plays an important role in the immune system, was previously sequenced from Japanese flounder (AB490772) and a microsatellite was identified in that region (Marker name: JFMHC1, Forward primer: GGCCTGGATAATGTGGACAC, Reverse primer: GAGTGTTGGGCCTTGGTG). The 5 S rRNA gene, which is a conserved component of the large ribosomal subunit, was isolated from Japanese flounder by [28] (AB154836-AB154839), and a microsatellite marker was isolated from the variable non-transcribed spacer (NTS) region (Marker name: JF5SrRNA, Forward primer: TGCACCTTGAGATTGATTTTGGAACA, Reverse primer: CACCCACAATACCTCCTTTCAGTCTT). Those microsatellites were also genotyped in the BACE family.

### SNP markers

SNP markers whose names begin with "AB" were derived from new EST sequences of *P. olivaceus*. RNA was isolated from Japanese flounder embryos, using a FastPure RNA kit (Takara Bio). cDNA strands were synthesized with the SuperScript Lambda System for cDNA Synthesis and Cloning (Gibco BRL) and then ligated to the derived phage ZIPLOX and packaged *in vitro *using the Packagene Gigapack III Gold (Stratagene). The phage vector was transformed to the plasmidial form by *in vivo *excision with *E. coli *DH10BZIP, following the manufacturer's instructions. Sequencing templates were prepared from positive clones using a TempliPhi DNA Amplification kit (Amersham Biosciences) and *Bca*BEST Primer RV-M 5'-GAGCG GATAA CAATT TCACA CAGG -3' (Takara Bio). Putative SNPs from the ESTs were prepared as described below.

SNP markers whose names begin with "H" and "Hzm" were developed from *P. olivaceus *ESTs registered in the GenBank database, 498 unique EST consensus sequences were selected for SNP discovery. PCR primer pairs were designed by DYNACLUST (DYNACOM Co.) and annealing temperature was 60°C for all primers. Reactions were performed using parent DNA (KP-BA and KP-CE) as follows: 30 s at 93°C; 30 s at 54-60°C and 45 s at 72°C. When single and clear PCR products were confirmed by electrophoresis with 1% agarose gel, they were purified with AMpure kits (BECKMAN). Subsequently, sequencing was carried out with a Big Dye terminator kit ver3.1 (ABI).

All contigs were screened for SNPs using the software Namihei (Mitsui Knowledge Industry) and 25 bp sequences just before SNP sites were used for SNaPshot primers. All putative SNPs were genotyped in the BACE family. Annealing temperature was 58°C for all PCR primers, PCR amplifications were performed and products were purified with AMpure kits (BECKMAN). Genotyping reactions were carried out in 10 μl reactions, using an ABI Prism SNaPshot ddNTP Primer extension kit (1 μl of SnaPshot mixture; 1 pM of primer; 10-50 ng of template PCR product) and the following thermal cycles: 25 cycles at 96°C for 10 s, 50°C for 5 s, 60°C for 30 s. SNaPshot products were treated with 1 U CAP (calf intestinal phosphatase) at 37°C for 1 h and the enzyme was heat-inactivated at 75°C for 15 min. Genotyping results were visualized on an ABI 3100 genetic analyzer.

### Annotation and synteny

EST sequences used for linkage mapping were used as queries for NCBI-BLASTX under default settings. Hits with *E*-values < 1 × 10^-4 ^were considered significant. The genome location information for the corresponding genes was obtained from *Tetradon nigroviridis *and *Danio rerio *ENSEMBL.

### Linkage analysis for map construction

Segregation data were considered independently for male and female in the BACE family. Marker genotypes were analyzed with LINKMFEX. Pairwise analyses were performed and markers were sorted in linkage groups at a minimum LOD score of 4.0. Double recombination events were checked with MapManagerQTX version 2.0 [50] and a final marker order was determined. Graphic map files were generated using MapChart version 2.2 [51].

Estimates of the differences in sex-specific recombination rates along the linkage groups were performed using the RECOMDIF application of LINKMFEX.

### Estimated Genome size

Genome sizes were estimated for the male and female maps by two different methods. First, Genome Estimation size 1 (Ge1) was calculated to account for chromosome ends by adding two times the average spacing of framework markers to the length of each linkage group [52]. Genome Estimation Size 2 (Ge2) was determined using method 4 of [53], in which the total length of the linkage groups is multiplied by the factor (m+1)/(m-1), where m is the number of framework markers on the linkage groups. The estimated genome size for each sex was taken as the average of the two estimates.

## Authors' contributions

CCS participated in microsatellite library construction, marker isolation and validation, microsatellite genotyping, linkage and recombination analysis and drafted the manuscript. KF contributed to microsatellite marker validation and genotyping, linkage and annotation/synteny analysis and reviewed the manuscript. AO took part in microsatellite genotyping and developing the linkage analysis pipeline. OH bred the experimental fish. TS helped with marker developing and project design. KM, IN, AF, TM and HO developed and genotyped SNP markers. KH sequenced the microsatellite libraries and MT analyzed sequence data and designed PCR primers. JK and YH provided laboratory facilities and supervised sequencing. NO conceived and overlooked the project and reviewed the manuscript. All authors read and approved the final manuscript.

## Supplementary Material

Additional file 1 **Microsatellites in the BACE map**. Linkage groups for males and females, marker names, primer sequences, GenBank accession numbers and annealing temperatures. Click here for file

Additional file 2 **SNPs derived from ESTs in the BACE map**. Linkage groups for males and females, SNPs ID, Blast top hit data, primer sequences, nucleotide-permutation sites of SNPs, SNP and flanking sequences. Click here for file

Additional file 3 **Female genetic map data**. Includes number of recombinant progenies, distance between markers and LOD scores. Numbered spreadsheets correspond to linkage group numbers. Click here for file

Additional file 4 **Male genetic map data**. Includes number of recombinant progenies, distance between markers and LOD scores. Numbered spreadsheets correspond to linkage group numbers. Click here for file

Additional file 5 **MapChart data**. Genetic map data, it can be used to recreate map figures. Click here for file

Additional file 6 **Annotation of SNPs in the BACE map against Tetradon and zebrafish, genomes**. SNPs ID, linkage groups in Japanese flounder, hypothetical proteins and their map positions of Tetraodon and zebrafish. Click here for file

Additional file 7 **Oxford plot comparing the linkage maps of Japanese flounder and Tetradon/zebrafish**. The observed correspondences of SNP markers derived from ESTs of Japanese flounder are indicated. Numbers indicate the number of Japanese flounder markers with NCBI-BLASTX hits to particular Tetradon and zebrafish chromosomes. Click here for file
